# Electrodeposited Superhydrophilic‐Superhydrophobic Composites for Untethered Multi‐Stimuli‐Responsive Soft Millirobots

**DOI:** 10.1002/advs.202302409

**Published:** 2023-06-08

**Authors:** Zhiqiang Zheng, Jie Han, Sinan Ozgun Demir, Huaping Wang, Weitao Jiang, Hongzhong Liu, Metin Sitti

**Affiliations:** ^1^ Physical Intelligence Department Max Planck Institute for Intelligent Systems 70569 Stuttgart Germany; ^2^ State Key Laboratory for Manufacturing Systems Engineering Xi'an Jiaotong University Xi'an 710054 China; ^3^ School of Mechanical Engineering Xi'an Jiaotong University Xi'an 710054 China; ^4^ Intelligent Robotics Institute School of Mechatronical Engineering Beijing Institute of Technology Beijing 100081 China; ^5^ Key Laboratory of Biomimetic Robots and Systems (Beijing Institute of Technology) Ministry of Education Beijing 100081 China; ^6^ Institute for Biomedical Engineering ETH Zurich Zurich 8092 Switzerland; ^7^ School of Medicine and College of Engineering Koç University Istanbul 34450 Turkey

**Keywords:** magnetic robotics, miniature robotics, multiple stimuli response, soft robotics, stimuli‐responsive materials

## Abstract

To navigate in complex and unstructured real‐world environments, soft miniature robots need to possess multiple functions, including autonomous environmental sensing, self‐adaptation, and multimodal locomotion. However, to achieve multifunctionality, artificial soft robots should respond to multiple stimuli, which can be achieved by multimaterial integration using facile and flexible fabrication methods. Here, a multimaterial integration strategy for fabricating soft millirobots that uses electrodeposition to integrate two inherently non‐adherable materials, superhydrophilic hydrogels and superhydrophobic elastomers, together via gel roots is proposed. This approach enables the authors to electrodeposit sodium alginate hydrogel onto a laser‐induced graphene‐coated elastomer, which can then be laser cut into various shapes to function as multi‐stimuli‐responsive soft robots (MSRs). Each MSR can respond to six different stimuli to autonomously transform their shapes, and mimic flowers, vines, mimosas, and flytraps. It is demonstrated that MSRs can climb slopes, switch locomotion modes, self‐adapt between air‐liquid environments, and transport cargo between different environments. This multimaterial integration strategy enables creating untethered soft millirobots that have multifunctionality, such as environmental sensing, self‐propulsion, and self‐adaptation, paving the way for their future operation in complex real‐world environments.

## Graphical abstract

Soft millirobots with reconfigurable morphology, multimodal locomotion, and multifunctionality are fabricated by electrodeposition. Induced gel roots between the hydrophilic hydrogel and the hydrophobic elastomer enable robust and permanent integration. The fabricated millirobot exhibits shape morphing and locomotion in response to six environmental stimuli, such as humidity, temperature, light, radio frequency heating, magnetic field, and chemical solvent.

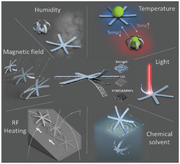

## 1 Introduction

In nature, numerous living organisms have evolved into remarkable heterogeneity to self‐transform their shapes or automatically change their motions to adapt to the local chemical and physical stimuli in their environments.^[^ ^1^ ^]^ Among these organisms, anisotropic components are the key to realizing unique properties, which respond to environmental stimuli and exhibit various behaviors or phenomena in nature.^[^ ^2^ ^]^ For example, to better adapt to complex and changing environments, many plants (e.g., sunflower,^[^ ^3^ ^]^ flytrap,^[^ ^4^ ^]^ mimosa,^[^ ^5^ ^]^ and pinecones^[^ ^6^ ^]^) can change the shape of their body parts, such as stems, leaves, flowers, and tendrils, in response to environmental stimuli (e.g., stress, light, temperature or humidity).^[^ ^7^ ^]^ These ingenious biological strategies provide inspiration for developing heterogeneous synthetic materials with unique structures and functions, accelerating the development of various applications, such as targeted cargo delivery, precision medicine, smart flexible electronics, and bionics.^[^ ^8^ ^]^

Compared with conventional robots, soft miniature robots often exhibit less dexterity and precision and poorer autonomous performance.^[^ ^9^ ^]^ These differences are attributable to their insufficient feedback mechanisms and lack of degrees of freedom (DOF) for different responses.^[^ ^10^ ^]^ To bring more capabilities of sensing and actuation to soft robots, many efforts have been made in the exploration of various stimuli‐responsive materials to perceive and react to environmental inputs,^[^ ^11^ ^]^ such as humidity,^[^ ^12^ ^]^ heat,^[^ ^13^ ^]^ light,^[^ ^14^ ^]^ chemicals,^[^ ^15^ ^]^ and magnetic fields,^[^ ^16^ ^]^ and convert input energy into mechanical energy output for self‐actuation,^[^ ^17^ ^]^ object manipulation,^[^ ^18^ ^]^ and object transport.^[^ ^7^, ^15^, ^19^ ^]^ It is important to develop suitable fabrication strategies that can tightly integrate multiple active materials or heterogeneous structures that can respond to various external stimuli to achieve higher performance and more advanced functions in the context of soft robots.^[^ ^20^ ^]^ By integrating various functions into intrinsically reconfigurable smart materials and structures, the soft robots would be able to sense multiple environmental stimuli, and in response, self‐change their shape and locomotion mode.

Inspired by natural vegetoanimal membrane structures, researchers have always combined a tough layer and a soft layer into a composite film for stronger interfaces between them.^[^ ^21^ ^]^ Based on this natural structure design, elastomers have been combined with xerogels to form a xerogel‐elastomer composite.^[^ ^22^ ^]^ The xerogel‐elastomer composite enables the xerogel and elastomer with different properties to complement each other, but the two materials are incompatible and require additional processes to combine them.^[^ ^23^ ^]^ To graft hydrogel polymers onto elastomeric surfaces, many chemical and physical methods exist, such as micro/nano‐architectural design on the interface for physical interlocking,^[^ ^24^ ^]^ plasma activation,^[^ ^25^ ^]^ spray coating,^[^ ^26^ ^]^ and structural soaking for coating free radicals and surface covalent crosslinking.^[^ ^27^ ^]^ However, natural biological components (e.g., alginate, chitosan, and proteins) cannot survive the chemical agents, high temperature, and pressure during these coating methods. Thus, we developed a method that first uses laser scribing to create laser‐induced graphene (LIG) as the conductive layer on elastomers with a natural nanoporous texture,^[^ ^28^ ^]^ and then furnishes stable adhesion to natural hydrogels and LIG‐coated elastomers under a mild reaction condition, which is so‐called electrodeposition. Electrodeposition combines physical interlocking and chemical ionic crosslinking and is thus fully sturdy.

**Figure 1:**
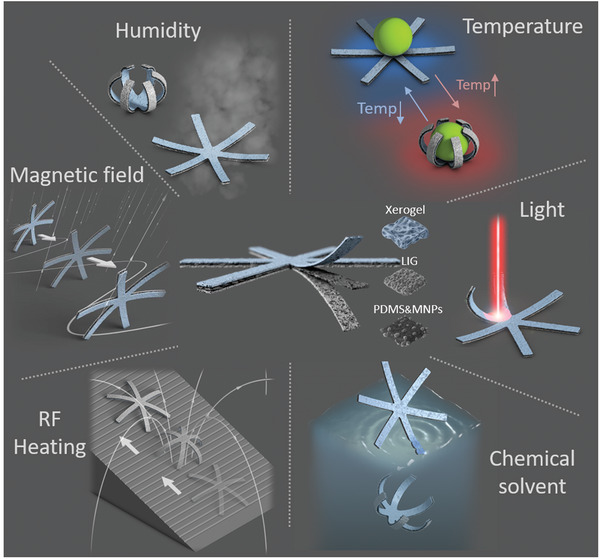
Schematic illustration of multi‐stimuli‐responsive soft robots (MSRs). MSRs have hydrogel (xerogel), elastomer (PDMS) and magnetic nanoparticle (MNP) composite sandwiched structures that enable shape morphing and locomotion in response to up to six stimuli: humidity, temperature, light, radio frequency (RF) heating, low‐frequency magnetic fields, and chemical solvents.

Here, we developed a fabrication strategy for quickly generating soft millirobots that are responsive to multiple stimuli, have reconfigurable structures, and can move in multiple ways. Our method involves combining 2D elastomeric sheets with a xerogel coating via electrodeposition‐based bonding. This approach generates gel roots between the superhydrophilic hydrogel and superhydrophobic elastomer through the LIG layer, forming a sandwich structure under normal pressure and temperature. The water‐free elastomers serve as a passive layer to support the actuator structure, while the xerogel acts as an active layer to produce the desired volume change in response to environmental stimuli. This sandwich structure can be laser‐cut into arbitrary shapes and act as a multi‐stimuli‐responsive soft robot (MSR), capable of changing shape and moving in response to six environmental stimuli: humidity, heat, light, radio frequency (RF) heating, low‐frequency magnetic fields, and chemical solvents. This versatility provides abundant degrees of freedom in control. To demonstrate the potential of our MSRs, we created a snowflake‐shaped robot that can move both in air and liquid, conduct self‐propulsion, climb slopes, self‐grasp, and self‐release, as shown in **Figure** **1**. Our reversible shape‐morphing MSRs offer potential applications in environmental sensing, self‐adaptation, soft actuation, and drug delivery.

## 2 Results

### 2.1 The Design and Fabrication of the Multi‐Stimuli‐Responsive Soft Millirobots

The MSR includes three main layers: the alginate xerogel, LIG, and magnetic elastomer. By introducing magnetic (e.g., NdFeB and Fe_3_O_4_) micro/nanoparticles into a polydimethylsiloxane (PDMS) elastomer matrix, differently shaped MSRs can respond fast to external magnetic fields and conduct diverse locomotion modes as shown in Figure 1. As illustrated in Figure S1, Supporting Information, the MSRs were prepared by electrodepositing a layer of alginate hydrogel onto the surface of a LIG layer. First, LIG was generated onto the surface of polyimide (PI) tape by laser scribing. Next, an uncured PDMS composite was poured onto the LIG layer, and a blade was used to control its thickness to ≈120 µm and generate a smooth surface. Then, the PDMS composite layer was peeled off together with the transferred LIG after curing at 85 °C for 6 h. The LIG layer was used as the electrode, and alginate was electrodeposited on the surface of the LIG layer. The water contact angle of the LIG‐coated PDMS was 146°(Figure S2, Supporting Information). The tensile strength of the sandwich film was tested with various PDMS and hard (NdFeB) magnetic microparticle (MMP) mass ratios (Figure S3, Supporting Information). Then, the alginate hydrogel was dried at room temperature for 24 h.

**Figure 2:**
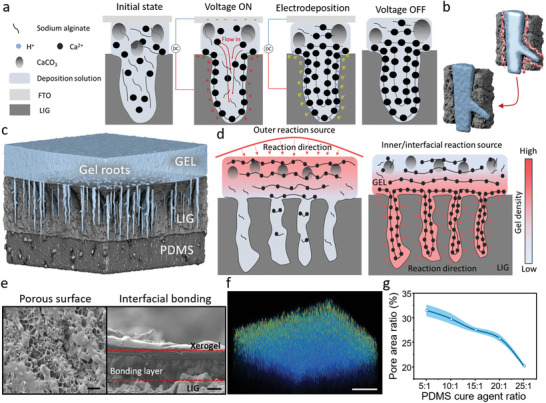
MSR fabrication process and sandwich structure integration. a) Schematics exhibiting the alginate hydrogel electrodeposition process in the porous LIG layer. The gel root is fabricated through inhomogeneous electrodeposition. b) The 3D schematics show the gelation process from the surface toward the center of the pore. c) Schematics demonstrated alginate hydrogel electrodeposition under different reaction sources. d) Illustration of the gelation process in the pore of the LIG layer. Two figures represent that the gel density always decreases along the reaction site. e) SEM images revealed the detailed LIG surface and the side‐view of the sandwich structure. The area between the red dash line is the adhesive layer. Scale bar: 5 µm. f) Fluorescent image shows the hydrogel interface structure. Scale bar: 100 µm. g) The pore area percentage depends on the PDMS cure agent ratio ± SEM with *N* = 3.

Based on the porous and conductive properties of the LIG layer, the detailed electrodeposition process in a single hole is shown in **Figure** **2**a. In the initial state, the space in the pore was filled with a deposition solution through the vacuum. By applying a constant electric current on both sides of the LIG layer, and fluorine‐doped tin oxide (FTO) plate, H^+^ ions and oxygen were produced by water electrolysis. During the electrolysis reaction, the H^+^ ions generated on the anode surface led to a rapid pH decrease and initiated the reaction for the deposition process. In the electrodeposition process, H^+^ reacted with CaCO_3_ particles to produce CO_2_ and Ca^2+^ ions which reacted with ‐COO^−^ on the sodium alginate to form the Ca‐alginate hydrogel. In this electrodeposition process, the electron exchange always occurs on the inwall of the porous surface of LIG, which can be regarded as an uneven electrode. Due to the mechanism of anodic electrodeposition of calcium alginate, the gel density always decreases from the anode to the cathode.^[^ ^30^ ^]^ Thus, in the pore structure, the gelation reaction was propagated from the inwall to the center of the pore (Figure 2b), which made the pore structure absorb unreacted components from outside the pore to form a tighter gel root. From a larger perspective, the grassy field‐liked sandwich structure utilized strong roots to firmly fix on to the ground (Figure 2c). In Figure 2d, the electrodeposition provided an inner/interfacial reaction source that directly occurred on the interface of two materials and formed the densest gel network in the interfacial region. In contrast to the outer reaction sources (e.g., heating, light, and cross‐linking agent), they always occurred on the outer surface of the hydrogel. Thus, the gel density decreased along the outside of the interface and formed the loosest gel network in the interfacial region.

To prove this mechanism, we characterized the surface of the LIG layer and the cross‐section structure of the interface between the gel and the LIG layer via scanning electron microscopy (SEM) and confocal microscopy (Figure 2e,f, and Figure S4 and Video S1, Supporting Information). The porous surface structure on the LIG layer was essential for the interfacial bonding, and the pore area ratio (regions with holes except for the whole region) of the LIG can be tuned by changing the PDMS cure agent concentration as illustrated in Figure 2g. Besides, we compared the LIG layer to other commonly used soft electrodes (such as a sprayed Ag layer and a sputtered Au layer on PDMS shown in Figure S5, Supporting Information). The sprayed Ag layer and the sputtered Au layer could not generate the grassroot‐like gel structure and bond with each other by electrodeposition because of the smooth surface, which proved the necessity of surface porosity. To compare with the outer reaction sources, we used a calcium chloride solution to crosslink the alginate hydrogel. But the crosslinked hydrogel was easy to be detached from the LIG‐coated PDMS in the washing process (Figure S6, Supporting Information). Thus, the outer reaction sources could not directly provide a constant bonding between the superhydrophilic and superhydrophobic materials. In general, the proposed electrodeposition process with a porous LIG‐based conductive layer provides a facile and efficient method to integrate superhydrophilic and superhydrophobic materials.

### 2.2 Intrinsic and Geometric Control of Shape Morphing

**Figure 3:**
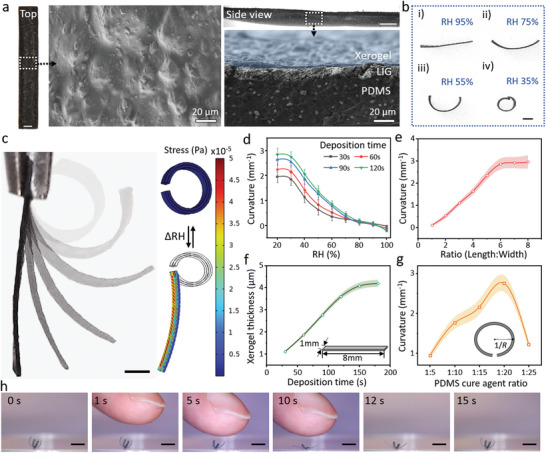
Controllable shape morphing of the electrodeposited hydrogel composite. a) Optical (left and top) and SEM (middle and right) images demonstrate a fabricated stripe structure. Top and cross‐section view SEM images of this fabricated stripe structure. Blue, gray, and black areas represent the xerogel, LIG, and PDMS layers, respectively. Scale bars: 500 µm. b) Time‐lapse images illustrated the stripe structure shape morphing process at 95% RH, exhibiting bending in response to a humidity change from 95% RH to 35% RH. Scale bar: 500 µm. c) Optical images (left) and bending simulations (right) of stripe structure transformation. Scale bar: 1 mm. d) RH level‐dependent bending curvature as a function of electrodeposition time varying from 30 to 120 s. Each dot represents the average curvature of three different stripe structures under an RH shift ± SEM. e) The length‐to‐width ratio of the stripe structures affects the bending curvature of the stripe structure after shape morphing. Each dot indicates ± SEM. with *N* = 5. f) xerogel thickness of the stripe microstructure formed with different electrodeposition times. Each dot represents the average xerogel thickness with stripe shapes ± SEM. with *N* = 3. g) PDMS curing agent ratio‐dependent curvature of stripe structure (crosslinker: prepolymer). Each dot represents the average curvature with stripe shapes ± SEM. with *N* = 5. h) Optical images of the cross‐shaped MSR sensing the human finger approaching it. Scale bar: 2 mm.

In **Figure** **3**a, the stripe‐shaped MSR was characterized by SEM, where the surface of the xerogel layer was observed to be rough and uneven. The cross‐section view exhibited a distinct sandwich‐like structure. A typical stripe structure showing a shape morphing process at different relative humidity (RH) levels is shown in Figure 3b and Video S2, Supporting Information. The experimental and simulation results of the stripe‐shaped MSR, shown in Figure 3c and Video S3, Supporting Information, prove that the control over bending extent could be realized by applying different RH levels. As shown in Figure 3d, the stripe‐shaped MSRs always achieve the largest curvature and remain in the bending state at low RH. The stripe‐shaped MSRs were in a straight form (open state) and even bent in the opposite direction when the RH was higher than 95%. In the ambient environment (25 °C and 30% RH), the MSRs were always in the open state and transfer to the closed state in rainy weather (25 °C and ≈70% RH). In the experiment with a series of different electrodeposition times, the MSRs showed the largest curvature in both the lowest (2.84 mm^−1^) and highest (−0.21 mm^−1^) RH levels at 25 °C under 120‐s electrodeposition as shown in Figure 3d. The resultant curvature can be expressed as *k* = 1/*r_min_*, where *k* is the curvature of the resultant structure and *r_min_* is the minimum radius of the spiral structure. The experimental results matched well with the simulation results.

Apart from the influence of humidity, the length–to–width ratio, PDMS cross‐linker concentration, and electrodeposition time were also vital factors influencing the shape‐morphing ability. Due to the shape‐morphing mechanism of the xerogel‐PDMS bilayer structure, the xerogel is the active layer that provided the stress of transformation in response to the humidity and temperature. When the width‐to‐length ratio was close to 1, the structure was transformed in a central symmetric manner as shown in Figure 3e. When there was a small width‐to‐length ratio (1:4), the structure was transformed in an axis‐symmetric manner. The curvature of the stripe structure experienced a constant increase when the width‐to‐length ratio increased from 1:1 to 1:6 and was maintained at 3 mm^−1^ when the ratio was 1:7 and 1:8. In addition, the relationship between the bending curvature, the thickness of the xerogel (active layer), and PDMS cross‐linker concentration (passive layer) is shown in Figure 3f,g.

There is a positive correlation between the electrodeposition time and the thickness of the xerogel layer. Initially, the thickness of the xerogel sharply increased with the deposition time from 30 to 120 s (voltage: 4 V). After 120 s, the thickness increased smoothly to 4.2 ± 0.1 µm when the deposition time was 180 s, which indicates that the gel thickness will reach a maximum value when the deposition time was over 150 s. As the PDMS mechanical properties were influenced by the weight ratio of the prepolymer and cross‐linker, different PDMS cross‐linker concentrations (weight ratio of the cross‐linker to prepolymer) were tested as illustrated in Figure 3g. In the same 150‐s electrodeposition time, the bending curvature of the stripe structure increased when the PDMS curing agent concentration decreased from 1:5 to 1:20, which was mainly due to the decrease in Young's modulus of the PDMS layer. However, when the PDMS curing agent ratio was decreased to 1:25, the PDMS layer became too soft to peel off and to transfer the LIG layer from the PI tape, which limited the electroconductivity of the LIG‐based electrode layer as well as the subsequent electrodeposition process. The MSR suffered a great performance reduction, and the bending curvature decreased from 2.7 ± 0.5 to 1.3 ± 0.3 mm^−1^. In the design of anisotropic deformation, the structure was always bent along the long axis. The cross‐shaped MSR could feel the humidity change under the close appearance of a finger by opening up in 10 s, and then reverting to the previous shape in 5 s when the finger was removed (see Figure 3h and Video S4, Supporting Information).

### 2.3 Bioinspired Shape‐Morphing Architectures

**Figure 4:**
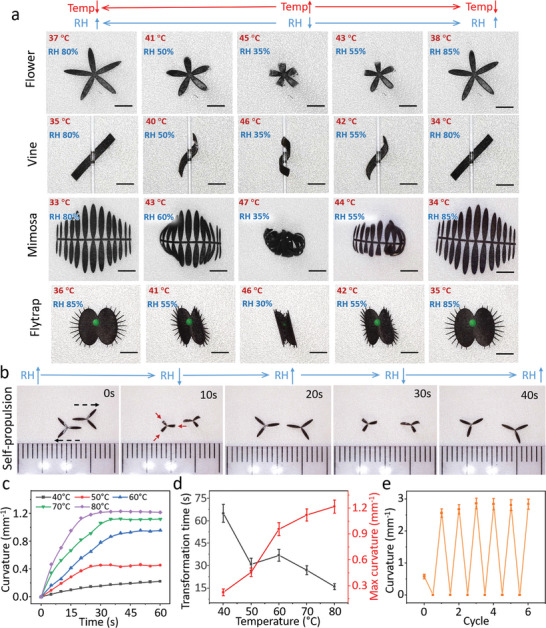
Bioinspired shape morphing demonstrations as a function of the environmental humidity and temperature changes. a) Various reversible movements of artificial vegetation, including flowers, vines, mimosas, and flytraps. Scale bar: 2 mm. b) Self‐propulsion of the asymmetric petal‐shaped structures via circular RH shift. c) Time‐dependent curvature as a function of environmental temperature varying from 40 to 80 °C. d) Temperature‐dependent transformation time (black line) and maximum curvature (red line). Each dot represents the average transformation time and curvature for three different stripe structures under a temperature shift with stripe shapes ± SEM. e) Transformation cycle of the stripe structure in RH range of ≈30–95%. Each dot represents the average curvature for five different stripe structures under an RH shift with stripe shapes ± SEM.

Besides a simple transformation from a stripe to a circle, this flexible fabrication method also allowed us to induce diverse deformation patterns by predesigning the structure shape. Inspired by the nastic movement of plants, such as flowers, vines, mimosa, and flytraps, bioinspired sheets were cut out as artificial vegetation (**Figure** **4**a and Video S5, Supporting Information). Due to the uniform shrinkage of the xerogel layer, we could design the local width‐to‐length ratio to control the anisotropic transformation of the structure. Since RH was negatively correlated to temperature, the swelling and shrinkage of xerogel could be tuned by both humidity and temperature. These artificial vegetations could lead to reversible shape morphing under cyclic RH and temperature changes. Figure 4b and Video S6, Supporting Information depict that asymmetric structures can conduct self‐propulsion in response to environmental RH changes. In the shape morphing process, the bending curvature and speed were largely affected by the temperature as shown in Figure 4c.

All tests were conducted at an RH range of 90% ± 3%. The stripe structures had the largest curvature of 1.2 mm^−1^ at 80 °C, while the stripe structures only bent slightly with a curvature of 0.21 mm^−1^ at 40 °C. Figure 4d demonstrates that a higher temperature always caused a smaller transformation time and larger curvature. For instance, the stripe structure only took 16 s to complete the transformation with a curvature of 1.26 mm^−1^ at 80 °C. For reversible shape morphing, the transformation cycle of the stripe structure is shown in Figure 4e. In this test, we changed the RH from 30% to 95% in more than five cycles, and the curvature exhibited a slight increase in the first two cycles and remained stable after the third cycle. Because the whole xerogel layer could not fully swell and absorb water from the air in the first two cycles, only the surface part of the xerogel provided volume shrinkage during the transformation. After two cycle transformation, the whole xerogel layer was fully moist and able to shrinkage in the low‐humidity and high‐temperature environment.

### 2.4 Robotic Shape Morphings Induced by Laser‐ and Radio Frequency Wave‐Based Remote Heating

**Figure 5:**
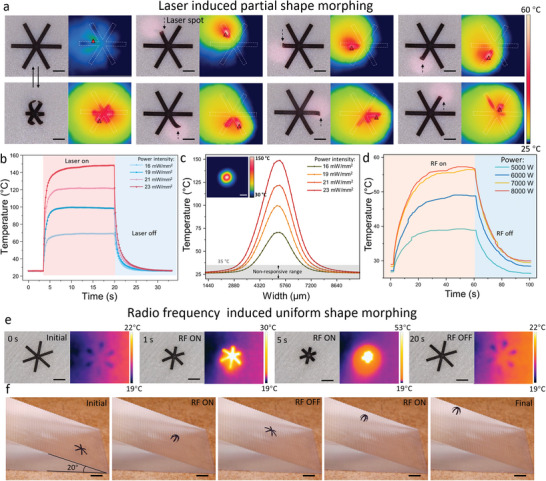
Programmable shape morphing demonstration in response to the laser‐ and RF wave‐based remote heating. a) Optical and thermal images of MSRs conducting partial shape morphing in response to laser heating. The dashed arrow points out the position of the laser spot. Scale bar: 500 µm. b) Time‐dependent temperature as a function of the power intensity that produces the laser varying from 23 to 16 mW mm^−2^. c) Width‐dependent temperature as a function of the power intensity that produces the laser varying from 23 to 16 mW mm^−2^. The thermal image gives the size and temperature value of the laser spot at 23 mW mm^−2^. Scale bar: 500 µm. d) Time‐dependent temperature as a function of the heating power that produces the alternating electric field (frequency: 300 kHz) varying from 5000 to 8000 W. e) Optical and thermal images of the MSRs conducting uniform shape morphing in response to the radio frequency heating. Scale bar: 1 mm. f) Optical images of the MSRs climbing the slope with a choppy surface. The transformation of the MSR was controlled by RF heating. Scale bar: 2 mm.

Besides responding to changes in humidity and direct heating, the MSRs could also conduct shape morphing in response to laser‐ and RF wave‐based remote heating as shown in **Figure** **5**. The snowflake‐shaped structures were taken as an example to show the self‐shape morphing ability. Figure 5a and Video S7, Supporting Information reveal that the MSRs could be controlled by a laser (wavelength: 808 nm) to conduct partial or uniform shape morphing. Each leg of the snowflake‐shaped MSRs could self‐bend in response to local laser heating at 90% RH, the response time of which is shown in Figure 5b. The minimum response temperature was ≈35–40 °C at 90% RH. Thus, the MSR could respond to the laser within 2 s and reach the maximum curvature after 2 to 7‐s exposure.

When the laser power intensity was 23 mW mm^−2^, the maximum heating temperature could reach 151 °C. After turning off the laser, the temperature rapidly decreased to room temperature within 5 s. In this heating process, the heat transfer efficiency was largely increased by the LIG (see Figures S7 and S8, Supporting Information). Figure 5c shows the temperature distribution of the laser spot. Because the minimum response temperature was 35 °C, the 16 mW mm^−2^‐powered laser had the smallest effective diameter (2.9 mm), which was the area that could trigger the transformation of the MSRs. The effective diameter of the 23 mW mm^−2^ powered laser was ≈4.9 mm. The LIG‐coated sandwich structure always had a larger and higher temperature range than the pure PDMS film. Based on these measurements, we could choose different laser powers to control the size of the effective area from the whole MSR body to a small segment of the MSR. If the MSRs moved into a concealed and lightproof site, the transformation of the MSRs could also be controlled by RF heating. The MSR thermal response to RF‐magnetic heating is shown in Figure 5d. A higher current always gave a better heating efficiency.

When the RF power was 7000 and 8000 W, MSR could respond to heating and conduct transformation in 2–3 s, and the highest body temperature of the MSR was ≈57 °C. After turning off the RF‐magnetic heating, the temperature rapidly declined to room temperature within 20 s. Due to the embedded Fe_3_O_4_ nanoparticles, the MSR could exhibit uniform shape morphing (Figure 5e and Video S8, Supporting Information) under a high‐frequency (375 kHz) magnetic field. Figure 5f and Video S9, Supporting Information demonstrate that the MSR was able to climb on a slope with a choppy surface with its front legs holding the front embossment during the closing process and the back legs engaging the back embossment during the opening process. The setup of the slope climbing is shown in Figure S9, Supporting Information.

### 2.5 Multimodal Locomotion of MSRs with Different Shapes in Various Environments

**Figure 6:**
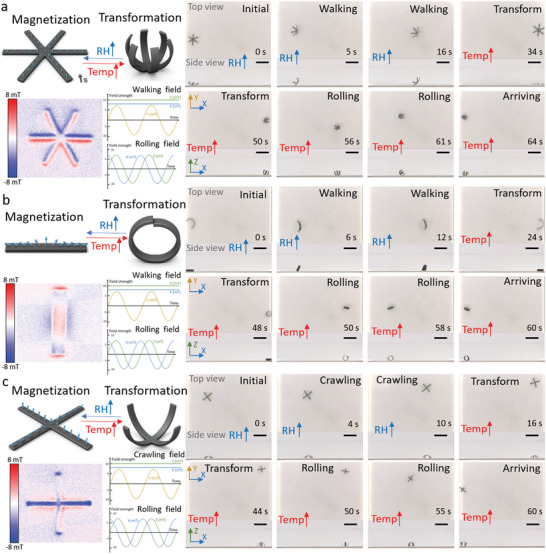
MSRs with different shapes conducting multiple locomotion modes to adapt to different environments. a) Schematic representation of the snowflake‐shaped MSR transformation, the pattern of magnetization moment, and the control signal of each motion (left) and snapshots (right) of its walking and rolling locomotion in moist and hot environments, respectively. Scale bar: 5 mm. b) Schematic representation of the stripe‐shaped MSR transformation, the pattern of magnetization moment, and the control signal of each motion (left) and snapshots (right) of its walking and tire‐like rolling in moist and hot environments, respectively. Scale bar: 5 mm. c) Schematic representation of the cross‐shaped MSR transformation, the pattern of magnetization moment, and the control signal of each motion (left) and snapshots (right) of its crawling and rolling in moist and hot environments, respectively. Scale bar: 5 mm.

Based on this flexible fabrication method, the MSRs could be fabricated in various arbitrary 2D shapes with a programmable magnetization moment as shown in **Figure** **6**. Figure S10, Supporting Information indicates the magnetic hysteresis curves (four‐quadrant BH curves) of the MSRs using various PDMS and MMP mass ratios. The locomotion of the MSRs was controlled by the external magnetic field. In addition to self‐shape morphing, the MSRs were able to automatically transform between different shapes and locomotion patterns to adapt to dynamically changing environments. In Figure 6a and Video S10, Supporting Information, the snowflake‐shaped MSR could walk in a moist and cool environment and roll in a hot and dry environment. The magnetic actuation signals of walking and rolling are shown in Figure 6a. The stripe‐shaped MSR could walk in a moist and cool environment and conduct wheel‐like rolling in a hot and dry environment (Figure 6b and Video S11, Supporting Information). The cross‐shaped MSR could crawl in a moist and cool environment and roll in a hot and dry environment (Figure 6c and Video S12, Supporting Information). All these MSRs moved from the left‐hand side under a high relative humidity of around 70% and 30 °C and moved from the right‐hand side with an ≈30% RH and 50 °C.

### 2.6 Untethered Snowflake‐Like MSRs with Locomotion and Self‐Gripping

**Figure 7:**
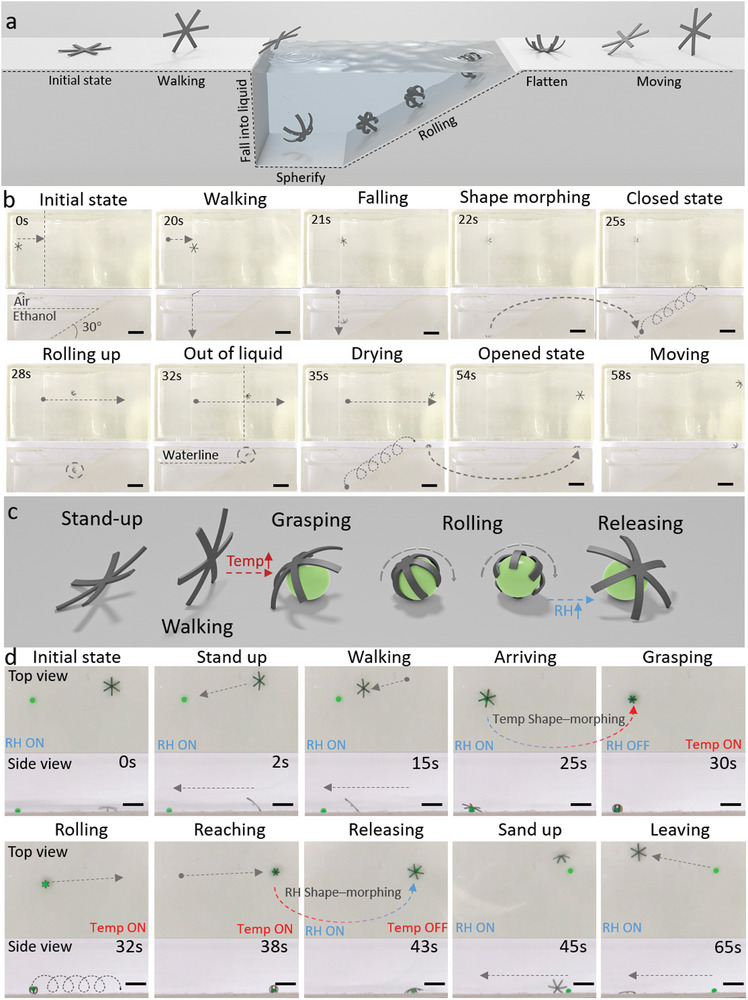
An untethered in situ reconfigurable MSR that can self‐adapt to different environments and terrains (air and liquid here) by exhibiting distinct locomotion modes. a) Schematic illustrations of different locomotion modes on a substrate in air and liquid exhibited by the MSR in response to external magnetic fields. b) Optical images from the top (top part in each row) and cross‐sectional (bottom part in each row) views of the MSR walking on a substrate in air and rolling by spherical structure inside a liquid. The shape morphing between these two locomotion modes is triggered autonomously when the MSR falls into the ethanol and the MSR can return to the flat sheet after moving out of the liquid. Scale bar: 6 mm. c) Schematics of different locomotion modes of the MSR, including walking, grasping, releasing, and rolling. The shifts between each motion were triggered autonomously by an increase in the environmental temperature and a decrease in the relative humidity. d) Optical images show the MSR walking to the target cargo, self‐grasping it (green ball diameter: 1 mm) in response to the environmental changes, transporting the cargo to the target area, releasing it, and retreating from the target area. Scale bar: 4 mm.

We evince an untethered MSR capable of multimodal locomotion with in situ shape reconfigurability in different working environments, that is, air and liquid, as shown in **Figure** **7**a. The locomotion of the MSR was controlled by the external magnetic field (Figure 6a). Being amphibious and motile on both dry surfaces as well as inside a liquid significantly widens the variety of future potential applications of the MSRs. To realize in situ reconfigurability during locomotion, the large shape‐morphing of the composite film was triggered by an external stimulus to self‐adapt the MSR body into shapes that were suitable for different locomotion modes in different environments and terrains. It remained flat on a substrate in the air at room temperature and 90% RH.

Under an external magnetic field, the MSR walked on the substrate and moved toward the substrate edge. The MSR kept walking until it fell into the liquid container (≥ 99% ethanol at room temperature). Considering that ethanol could absorb water from the MSR, the MSR induced shape‐morphing to form an overall spherical shape. Once the desired spherical shape was formed, the magnetic field was rotated in the vertical plane, which induced the rolling of the MSR. We controlled the MSR to move on the slope to showcase its motility. Once the MSR moved out of the liquid, the adsorptive ethanol evaporated in a short time, and the MSR could absorb water from the ambient environment at the same time, resulting in a flat shape. After the complete evaporation of adsorptive ethanol, MSR could walk as shown in Figure 7b and Video S13, Supporting Information.

Figure 7c,d and Video S14, Supporting Information illustrate that, based on the untethered reconfigurable MSR, we could grasp the target object/cargo and deliver it to the target area with multimodal locomotion in response to different environmental stimuli. The MSR remained flat on a substrate in air at room temperature and 90% RH. During this process, the robot was controlled as shown in Figure 6a. The robot was magnetized along the vertical direction in its open state. Then, we provided a uniform field along the *Z‐* and *X‐*directions (*B_Z_* > *B_X_*). Thus, the robot could aslant stand up. Meanwhile, we provided an oscillating field along the *Y‐*axis, and the robot could vacillate to the left and to the right. Because the robot was always tilted along the *X‐*axis, the robot moved along the *X‐*axis. The MSR was able to cover the target by adjusting the posture and direction of the MSR, and perfectly grasped the target in 2–3 s, which was triggered by a different external stimulus (temperature and humidity). After object grasping, the MSR was capable to carry it rolling to the intended area and then implementing release and retreat. The MSR could grasp and carry at least 2.5 mg objects under 45 °C and RH 30%. The change from a “sheet” to a “sphere” shape shown here is drastically different from the previously shown shape‐morphing demonstrations and enables very different locomotion modes in two media with vastly different viscosities, that is, air and ethanol.

## 3 Conclusion

In this study, we combined laser scribing and electrodeposition to permanently bond the superhydrophilic hydrogel onto the superhydrophobic LIG‐coated PDMS. We found that electrodeposition is an efficient and simple method to firmly combine these materials under mild reaction conditions. The resulting MSRs could undergo a fast and large degree of shape transformation, providing actuation, propulsion, and self‐adaptability to the MSRs in response to six different environmental stimuli: humidity, light, temperature, RF, magnetic field, and chemical solvent. We were able to fabricate customizable 2D‐shaped architectures with various patterns of shape morphing with a minimum width of 200 µm. The bending force of the MSR was tested in Figure S11, Supporting Information. Our MSRs could accomplish diverse tasks, such as self‐propulsion, slope climbing, and cargo transportation and release. These MSRs can be used in future applications in complex terrains where rolling and walking gait can be used depending on the environment, and shape transformation can be controlled by light or RF‐magnetic heating when the environment is lightproof or concealed.

Our proposed electrodeposition method allows for the integration of superhydrophilic and superhydrophobic materials to generate multi‐stimuli‐responsive soft millirobots that have numerous potential applications in soft actuation, cargo delivery, and environmental sensing. The multi‐stimuli‐responsiveness of our MSRs provides more sensing and movement abilities, enabling them to adapt to the diverse local environment. This provides possibilities for developing physically intelligent millirobotic systems^[^ ^29^ ^]^ and providing more precise control and sophisticated functionalities in the future.

## 4 Experimental Section

### Laser Scribing Parameters

A 150 W CO_2_ laser cutter/engraver (wavelength: 10.6 µm, beam size ≈120 µm, Universal Laser System, PLS6‐150D) was used to generate the conductive layer of LIG onto PI tape (Kapton®, Electron Microscopy Sciences). The laser engraving process for LIG production was operated under ambient conditions with the PI tape attached to a 2‐mm thick glass plate, and the optimized parameters were a power of 4.9 W, speed of 450 mm s^−1^, points per inch of 1000, and raster mode. Then, the uncured PDMS and NdFeB hard magnetic microparticles (5 µm in average diameter, Magnequench GmbH, MQFP‐15‐7) were mixed in a 1:1 weight ratio by a planetary mixer (KK‐250S, Mazerustar) for 90 s and poured on the top of the graphene surface. The thickness of this magnetic sandwich structure was controlled to 120 µm by the thickness of spacer tapes, and the extra composite on the top was removed by a single‐edge razor blade, which also smoothed the top surface. After curing at 85 °C for 6 h, the LIG layer was transferred to the magnetic PDMS layer from the PI tape, and it could be easily pulled off together with the magnetic PDMS layer. The sheet resistance of LIG (before peeling off) was 71.528 Ω per sq (std = 1.365). The sheet resistance after peeling off was 254.268 Ω per sq (std = 4.170). A high‐resolution UV laser system (ProtoLaser U3, LPKF Laser & Electronics, wavelength: 355 nm, beam size: 20 µm, accuracy: 2 µm, repeatability: ±2 µm) was used for cutting the samples/sandwich structure the authors prepared into the desired 2D shape. The laser cutting process was operated in ambient conditions with the sandwich‐like sheet placed on top of a glass substrate and the xerogel layer placed face up.

### Electrodeposition Process

An electrodeposition solution containing 1% (w/v) sodium alginate and 0.75% (w/v) CaCO_3_ particles was utilized to create a xerogel layer. First, a 5 mL electrodeposition solution was deposited between two electrodes onto the anode plate, and vacuum the plate to fill in the nanopores with the solution for 5 min. Second, for 30–150 s, a direct current voltage of 3–5 V was applied to both ITO layers of the electrodes. Before detaching the hydrogel microstructures, the anode plate was washed with deionized water in a Petri dish for over 2 min after the electrodeposition process until the un‐crosslinked calcium alginate hydrogel microstructures were fully detached from the film. Finally, the anode plate was dried in a dark and damp place for 1 day. Before drying, the thickness of the alginate hydrogel was ≈200–800 µm, which was measured by scanning the entire structure under the confocal microscope (A1, Nikon, Japan). After drying, the thickness of the alginate hydrogel was ≈1–4 µm, which was measured by SEM imaging.

### Bending Experiments

Measurements were carried out in a custom humidity chamber with a small water atomizer and an RS Pro RS‐3322 humidity sensor. An Espec SH‐222 temperature/humidity chamber was used for the static measurements. Contact heating was carried out by a hot plate (Fisherbrand Isotemp Hotplate). Laser heating was conducted by a fiber‐coupled laser (BWF1‐808‐450‐E, 808 nm, 0–450 mW). RF‐magnetic heating was carried out by an induction heating system (Ambrell Corporation, US). The actuation responses of the samples were recorded with a camera setup (TOOLCRAFT USB microscope) and examined by extracting single frames from the videos. These frames were used to measure the curvature by ImageJ and assemble the superimposed figures. The temperature was measured with a FLIR‐A300 camera (FLIR Systems Inc.). The stability test was conducted on the MSR, and there was no delamination even in 95% RH for 12 h as shown in Figure S12, Supporting Information.

### Electromagnetic Control

A uniform magnetic field with a maximum strength of 11 mT was generated by a coil setup within a workspace with a size of 4 × 4 × 4 cm^3^ as shown in Figure S13, Supporting Information. The magnetic field was controlled by modulating the currents in the electromagnetic coils using six independent motor driver units (SyRen25) and an Arduino microcontroller operating at 1.2 kHz. The robot's motion was tracked using two cameras (Basler, aCa2040‐90uc). The first camera operated at 120 frames per second (fps) with a frame size of 2040 × 1020 pixels and was placed orthogonal to the *Y*–*Z* plane of the workspace to observe the robot from the side. The second camera operated at 60 fps with a frame size of 2040 × 1400 pixels and had a top view of the workspace through a mirror placed at an angle of 45° above the test surface.

### Finite Element Analysis

To obtain the simulation results, the commercial finite element analysis software Abaqus was used. The shape morphing of the structures was calculated as follows.

(1) $$m=\frac{h1}{h2}\;n=\frac{E1}{E2}$$

where *h*_1_ and *h*_2_ are the thicknesses of the PDMS and xerogel, while *E*_1_ and *E*_2_are Young's modulus of the PDMS and xerogel, respectively. The thickness of the PDMS and xerogel were 0.12 and 0.006 mm, respectively, which were measured from the SEM images. The elastic moduli of PDMS and xerogel were set as 0.8 MPa and 3.75 GPa, respectively, which were evaluated by the experimental test.

(2) $$1/\rho =\frac{6\left(\alpha_{2}-\alpha_{1}\right)\left(RH-RH_{0}\right){\left(1+m\right)}^{2}}{h\left(3{\left(1+m\right)}^{2}+\left(1+mn\right)\left(m^{2}+\frac{1}{mn}\right)\right)}$$

where *ρ* is the radius of curvature, *α*_2_ and *α*_1_ are the hygroscopic expansion coefficients of the xerogel and PDMS, respectively, *RH* and *RH*_0_ are humidity and reference humidity, respectively, and *h* is the total thickness of the materials. The coefficients of hygroscopic expansion coefficient of the xerogel and PDMS were 200 and 1, respectively. The Poisson's ratio of the printed strip was set to 0.49. Based on this model, the shape‐morphing results of various structures could easily be predicted.

## Conflict of Interest

The authors declare no conflict of interest.

## Author Contributions

Z.Z. and J.H. contributed equally to this work. Z.Z., J.H., S.O.D., and M.S. conceived the idea and designed the research. Z.Z., J.H., and S.O.D. constructed the experimental platform. Z.Z., J.H., and H.W. performed the research and analyzed the data. Z.Z. and J.H. formulated and implemented the computational model. M.S. supervised the research. Z.Z., J.H., H.W., S.O.D., W.F., H.L., and M.S. wrote the paper with contributions from all authors. All authors contributed to the discussion and wrote the manuscript.

## Supporting information

Supporting Information

Supplemental Video 1

Supplemental Video 2

Supplemental Video 3

Supplemental Video 4

Supplemental Video 5

Supplemental Video 6

Supplemental Video 7

Supplemental Video 8

Supplemental Video 9

Supplemental Video 10

Supplemental Video 11

Supplemental Video 12

Supplemental Video 13

Supplemental Video 14

## Data Availability

The data that support the findings of this study are available in the supplementary material of this article.
