# Supplementary material for: Electrodeposited Superhydrophilic‐Superhydrophobic Composites for Untethered Multi‐Stimuli‐Responsive Soft Millirobots
Source: Adv Sci (Weinh). 2023 Jun 8;10(23):2302409. doi: 10.1002/advs.202302409 (PMC10427389; doi:10.1002/advs.202302409)
Supplement: Supplementary file 1 — Supporting Information [file ADVS-10-2302409-s005.pdf]

ADVANCED SCIENCE
Open Access

Supporting Information

for *Adv. Sci.*, DOI 10.1002/advs.202302409

Electrodeposited Superhydrophilic-Superhydrophobic Composites for Untethered Multi-Stimuli-Responsive Soft Millirobots

*Zhiqiang Zheng, Jie Han, Sinan Ozgun Demir, Huaping Wang, Weitao Jiang, Hongzhong Liu and Metin Sitti**

Supporting Information

Electrodeposited superhydrophilic-superhydrophobic composites for untethered multi-stimuli-responsive soft millirobots

Zhiqiang Zheng, Jie Han, Sinan Ozgun Demir, Huaping Wang, Weitao Jiang, Hongzhong Liu, Metin Sitti

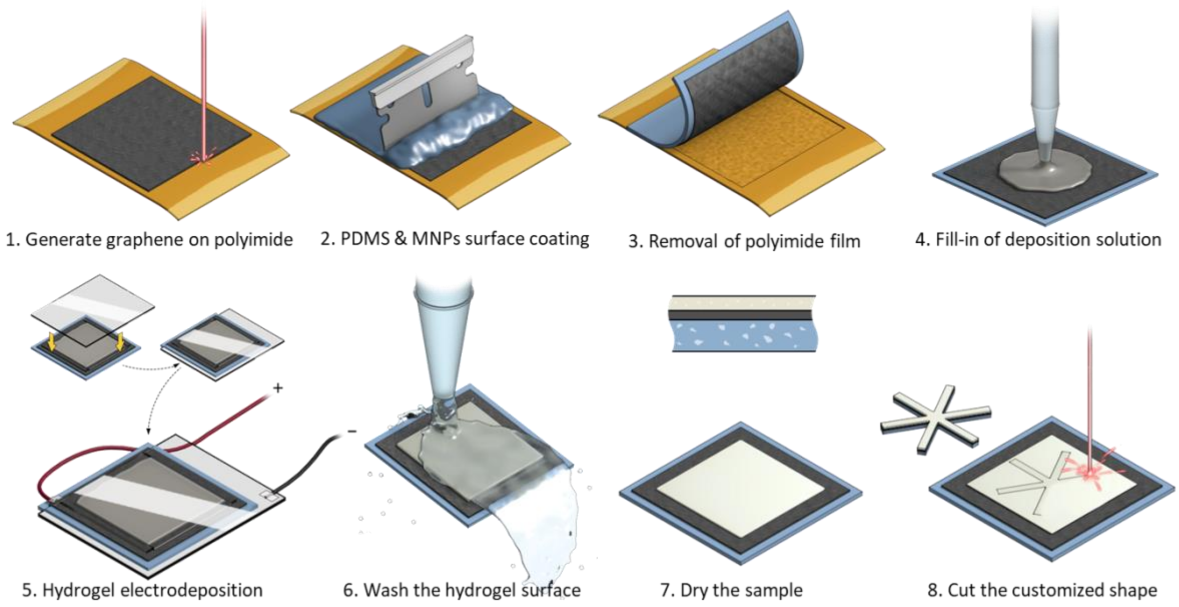

Figure S1. Schematics of the MSR fabrication process.

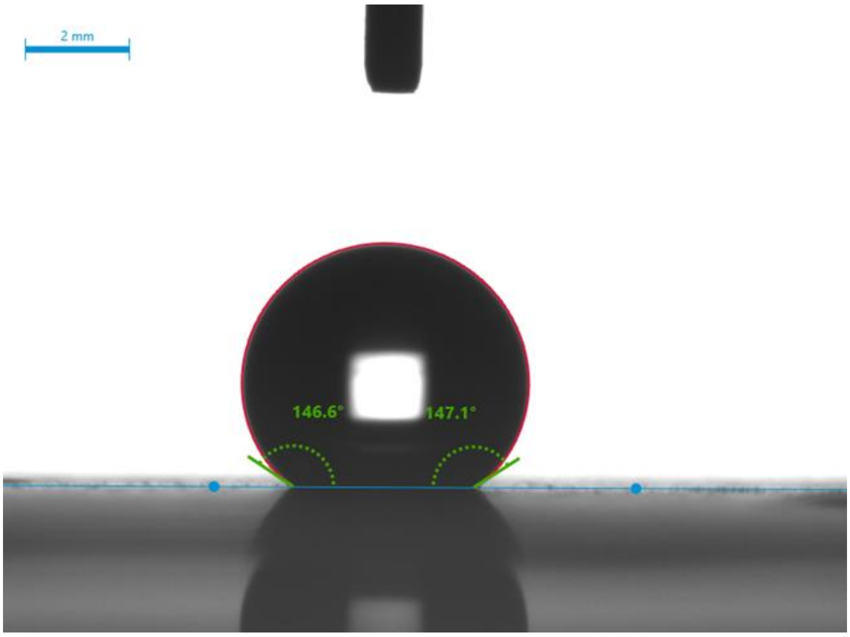

Figure S2. Water contact angle of the LIG-coated PDMS. 4 μL water droplet was used to test the contact angle of the surface.

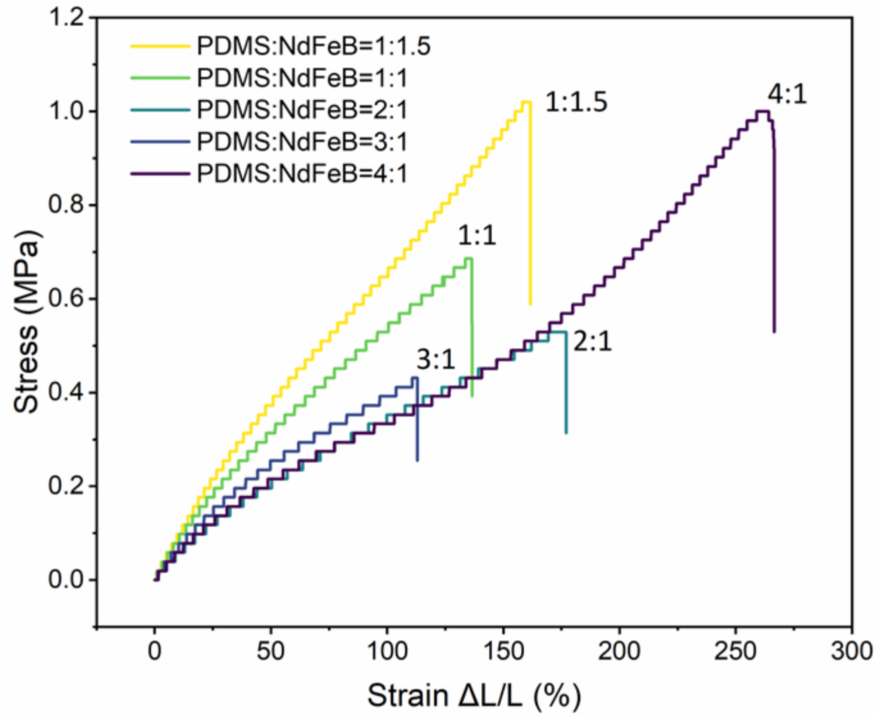

Figure S3. Tensile test of the composite stripe structures with PDMS and NdFeB microparticle ratio of 1:1.5, 1:1, 2:1, 3:1 and 4:1.

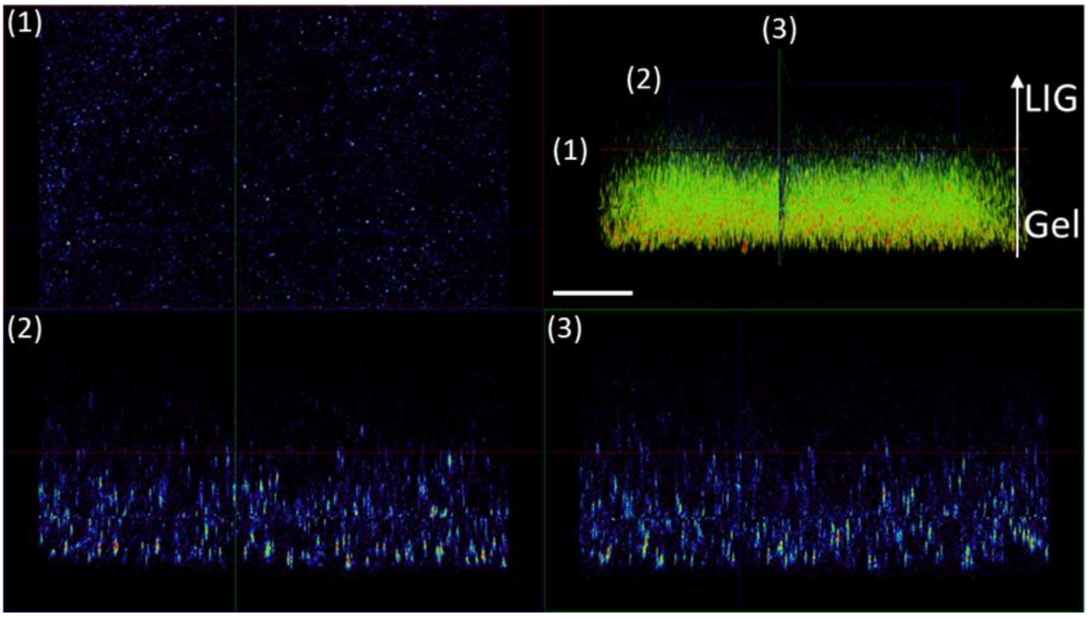

Figure S4. The three section views fluorescent images of hydrogel interface. This image is scanned along the surface of hydrogel to the LIG. Scale bar: 100 μm.

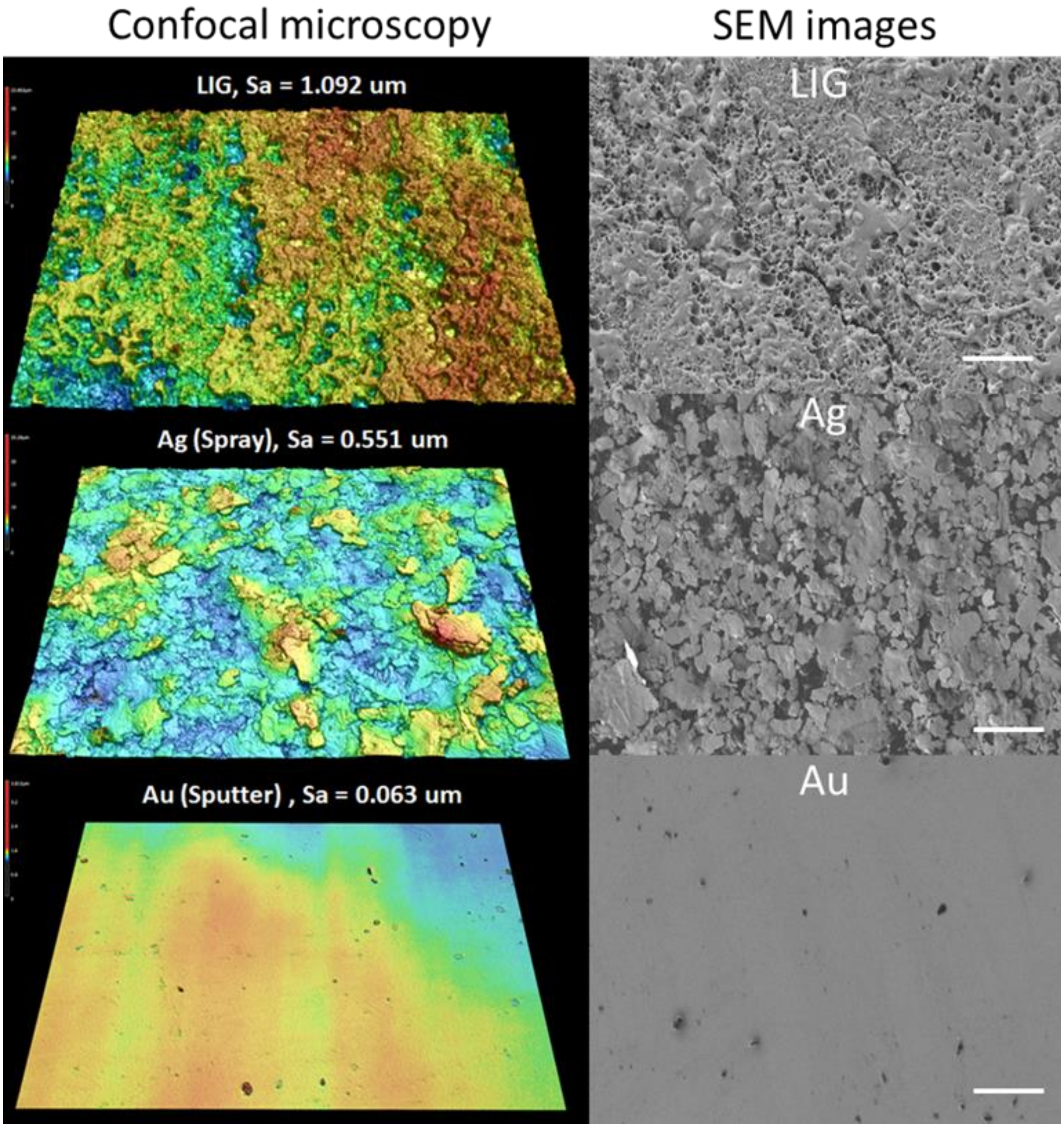

Figure S5. The confocal and SEM images of LIG, sprayed silver (Ag), and sputtered gold (Au) surface. Sa represents the arithmetical mean height of the surface. Scale bar: 20 µm.

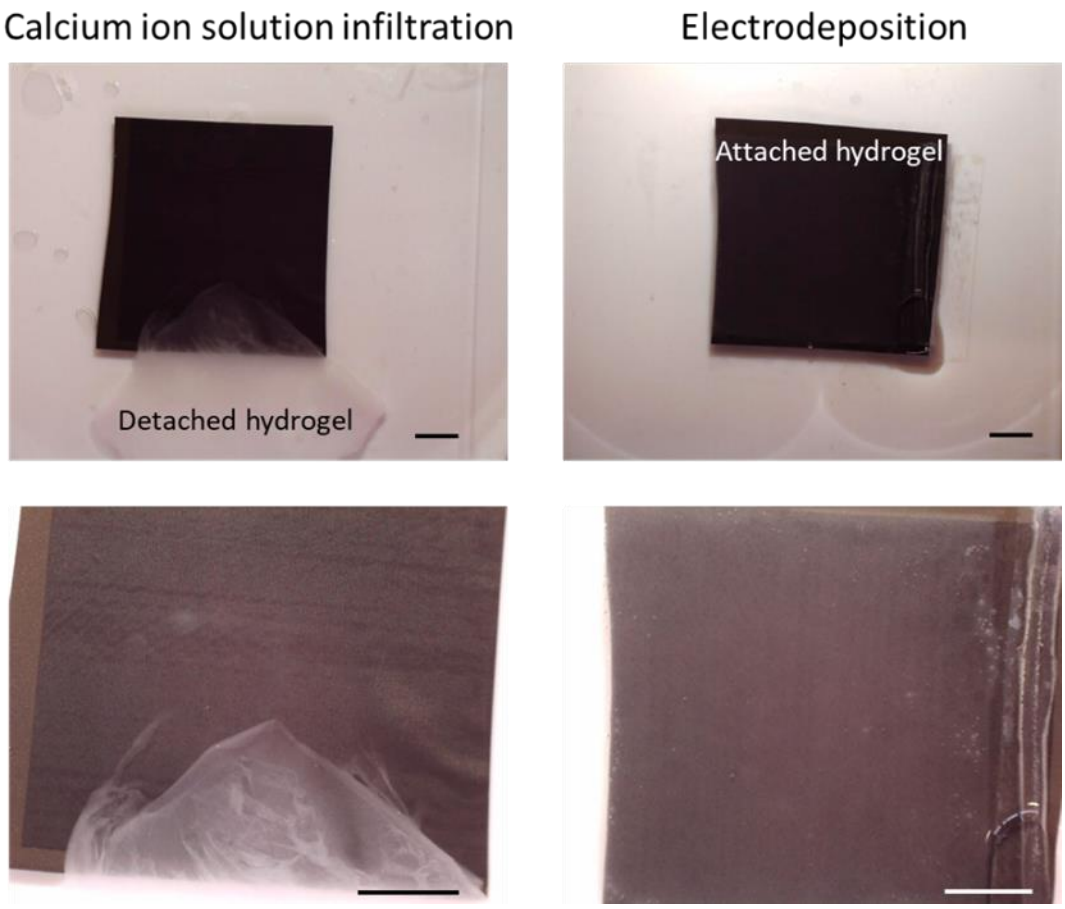

Figure S6. Calcium ion solution and electrodeposition gelation result after 1-min washing process. Scale bar: 1 cm.

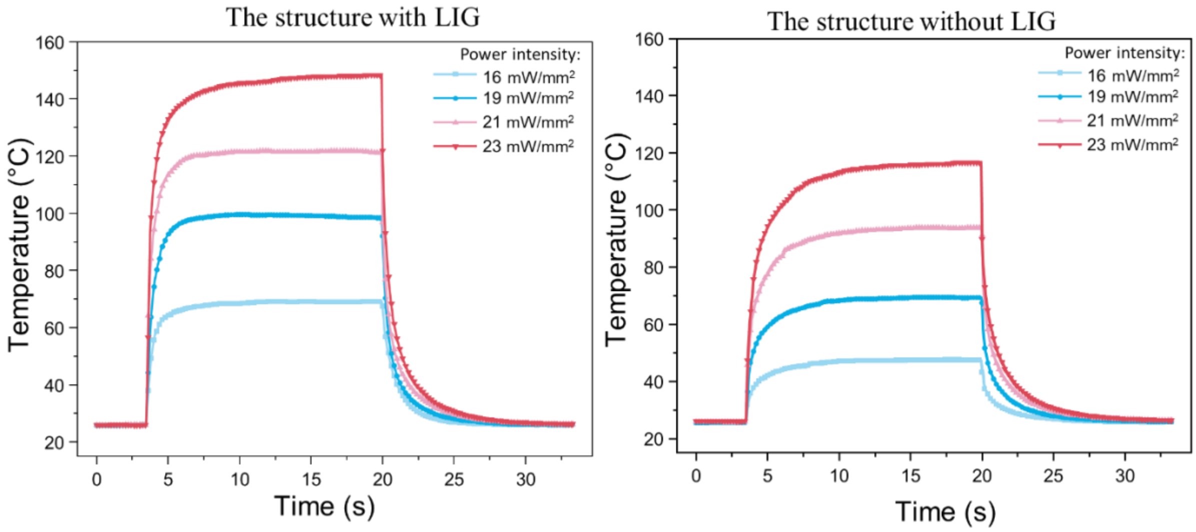

Figure S7. Time-dependent temperature as function of the power intensity. Power intensity produced by laser varying from 23 mW/mm^2 to 16 mW/mm^2 on PDMS film with and without LIG.

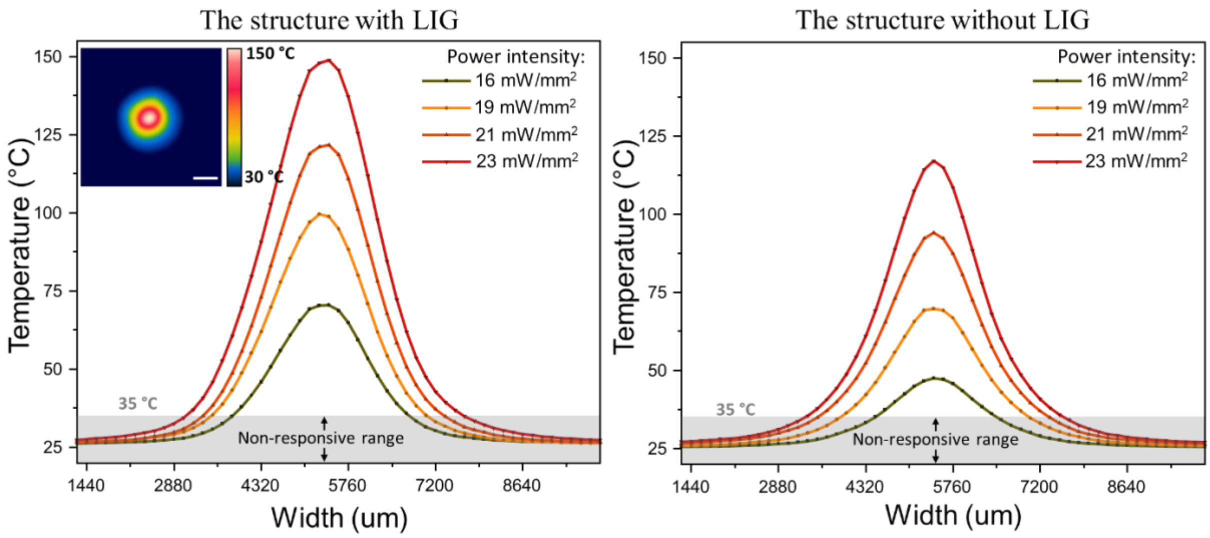

Figure S8. Width-dependent temperature as a function of the power intensity. Power intensity produced by laser varying from 23 mW/mm^2 to 16 mW/mm^2 on PDMS film with and without laser induced graphene.

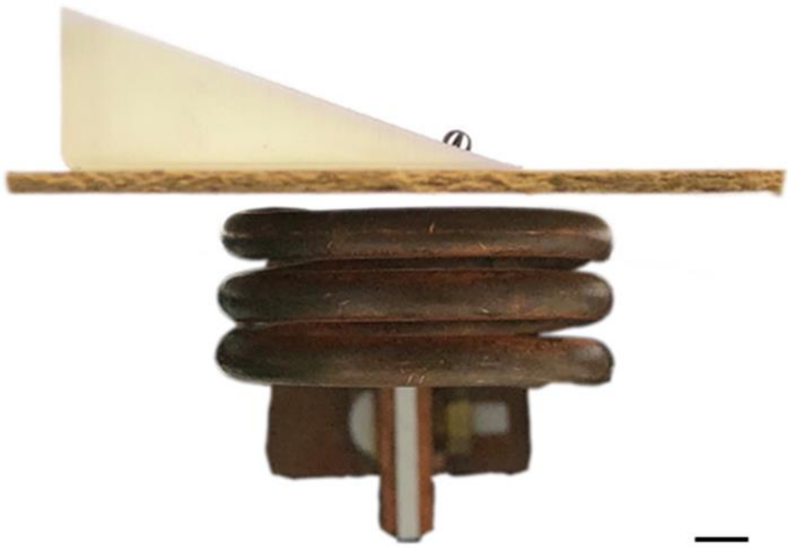

Figure S9. Photo of the radio frequncy (RF) magnetic field heating setup. Scale bar: 2 mm. The stage is around 1 mm above the electromagnetic coil.

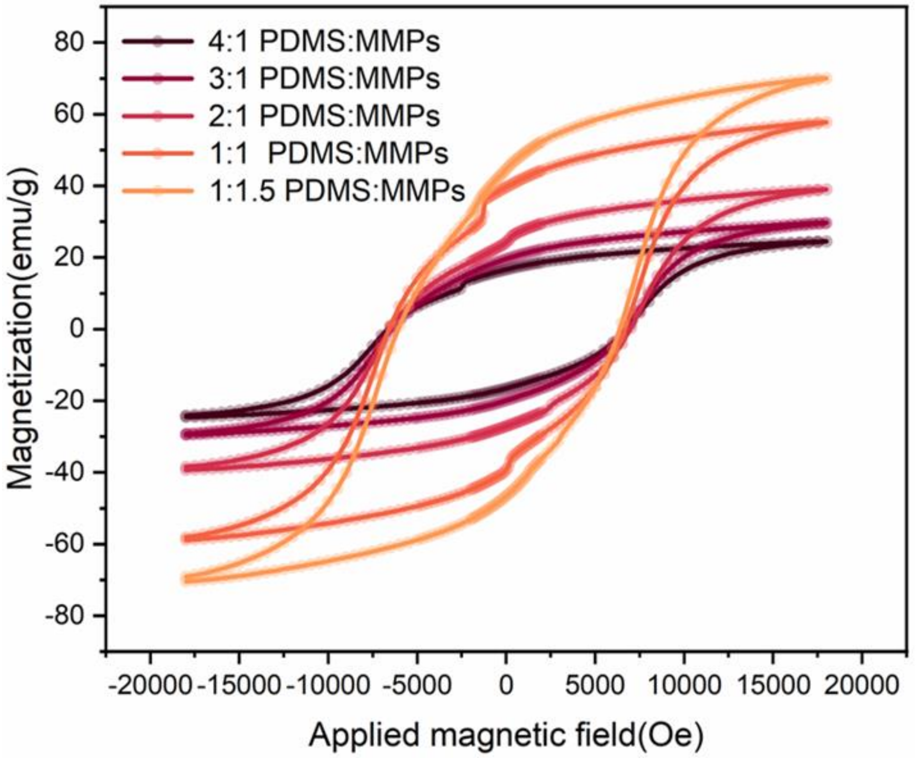

Figure S10. Measured magnetic hysteresis curves (four-quadrant BH curves) of the composite materials. The ratios are 4:1, 3:1, 2:1, 1:1, 1:1.5 of PDMS: magnetic microparticles (MMPs) mass ratio.

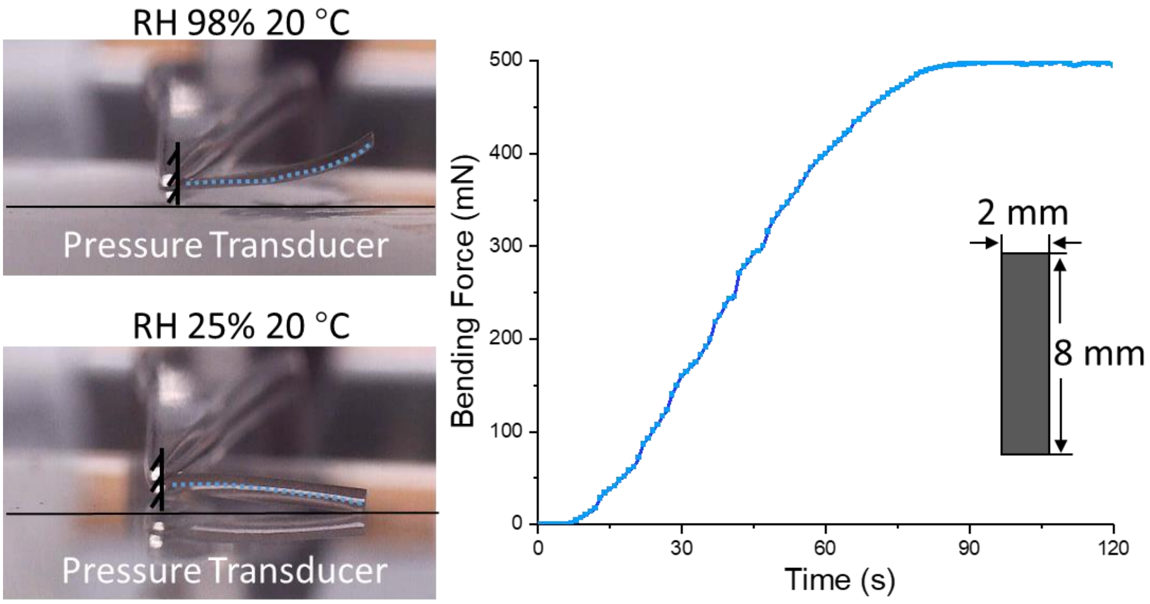

Figure S11. Bending force test of the MSR from 98% RH to 25% RH in the room temperature. The size of tested beam structure is 2-mm width and 8-mm length. The maximum bending force is around 500 mN.

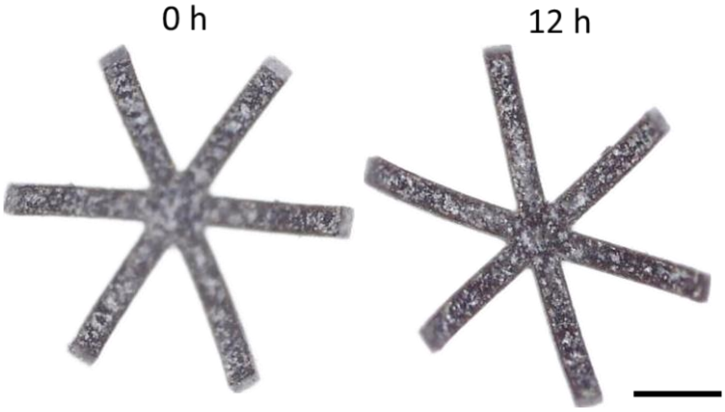

Figure S12. Stability test of the MSR in 95% RH for 12 hours. Scale bar: 1 mm.

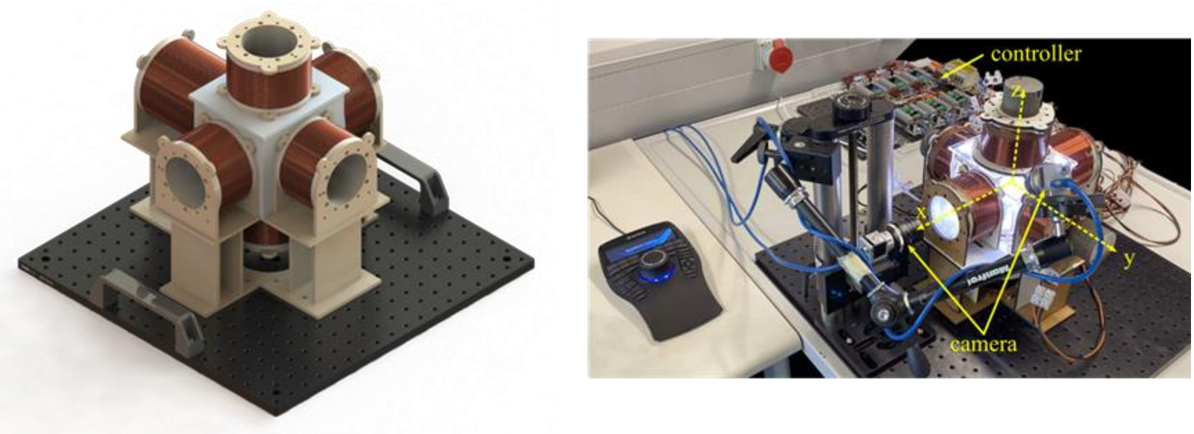

Figure S13. The schematic and image of the magnetic coil setup, which is used to apply 3D magnetic fields on the MSRs in the workspace.

Supplementary Videos

Video S1. Confocal microscopic 3D image of fluorescent nano particle loaded hydrogel interface.

Video S2. Bending of stripe structure in response to humidity change.

Video S3. Shape-morphing and simulation of the stripe structure.

Video S4. Hand humidity reaction of the cross structure.

Video S5. Shape-morphing of the flower, vine, mimosa and flytrap shaped structure

Video S6. Self-propulsion of asymmetric petal-shaped structures.

Video S7. MSRs conducting local transformation in response to the laser heating.

Video S8. MSRs conducting uniform shape morphing in response to the radio frequency heating.

Video S9. MSRs climbing the slope with choppy surface.

Video S10. Multimodal motion of snowflake shaped MSR in response to the environmental changes.

Video S11. Multimodal motion of stripe shaped MSR in response to the environmental changes.

Video S12. Multimodal motion of cross shaped MSR in response to the environmental changes.

Video S13. Movement adaptability of MSR in air and liquid.

Video S14. Multi-tasking of MSR in response to the environmental changes.
